# Pseudogene PHBP1 promotes esophageal squamous cell carcinoma proliferation by increasing its cognate gene PHB expression

**DOI:** 10.18632/oncotarget.16196

**Published:** 2017-03-16

**Authors:** Feiyue Feng, Bin Qiu, Ruochuan Zang, Peng Song, Shugeng Gao

**Affiliations:** ^1^ Department of Thoracic Surgery, National Cancer Center, Cancer Hospital, Chinese Academy of Medical Sciences and Peking Union Medical College, Beijing 100021, China

**Keywords:** natural antisense transcript (NAT), long noncoding RNA (lncRNA), esophageal squamous cell carcinoma (ESCC), quantitative real-time PCR (qRT-PCR), SDS-polyacrylamide gel electrophoresis (SDS-PAGE)

## Abstract

Natural antisense transcripts (NATs) as one of the most diverse classes of long noncoding RNAs (lncRNAs), have been demonstrated involved in fundamental biological processes in human. Here, we reported that human prohibitin gene pseudogene 1 (*PHBP1*) was upregulated in ESCC, and increased *PHBP1* expression in ESCC was associated with clinical advanced stage. Functional experiments showed that *PHBP1* knockdown inhibited ESCC cells proliferation, colony formation and xenograft tumor growth *in vitro* and *in vivo* by causing cell-cycle arrest at the G1-G0 phase. Mechanisms analysis revealed that *PHBP1* transcript as an antisense transcript of *PHB* is partially complementary to *PHB* mRNA and formed an RNA-RNA hybrid with *PHB*, consequently inducing an increase of *PHB* expression at both the mRNA and protein levels. Furthermore, *PHBP1* expression is strongly correlated with *PHB* expression in ESCC tissues. Collectively, this study elucidates an important role of *PHBP1* in promoting ESCC partly via increasing *PHB* expression.

## INTRODUCTION

In the past decades, esophageal squamous cell carcinoma (ESCC), as the most common type of esophageal cancer, has been one of the most leading causes of cancer-related death worldwide [1, 2]. According recent statistical data, incidence and mortality rate of ESCC is increasing rapidly occurring in China and the incidence of ESCC is around three times more common in men than in women [3, 4]. Though the improvements in the treatment of ESCC have been achieved by radiochemotherapy and surgical resection, its prognosis remains disappointed [5–7]. Therefore, fully understanding the genetic and molecular mechanism of ESCC development and progression is urgent for us to develop potential diagnostic and treatment approaches on ESCC.

Long non-coding RNAs (lncRNAs) represent a diverse type of long RNA molecules lacking protein-coding capacity, with a length of larger than 200 nucleotides [8, 9]. A plenty of evidence has proved that lncRNAs play essential roles in fundamental biological processes, such as cell growth, differentiation, immune response and cancer biology [10–19]. Recently, one group of lncRNAs is the natural antisense transcripts (NATs), accounting for about 50-70% of lncRNAs, transcribed from the opposite DNA strand of their endogenous sense counterpart's protein-coding genes and non-protein-coding genes [20, 21]. For many years, the well-defined transcriptional units are initially overlooked due to low levels of expression, and unknown functions. However, recently, antisense lncRNAs (aslncRNAs) have garnered increased attention due to their highly locus-specific effects. Several studies have revealed the critical roles of aslncRNAs in various pathophysiological processes, particularly in multiple diseases and cancers [22–25]. One major emerging theme is centered on the effects of aslncRNAs exerting *in cis* on their neighboring genes or *in trans* on other distant genes through transcriptional or post-transcriptional regulation [26, 27].

Human prohibitin gene (*PHB*) pseudogene 1 (*PHBP1*) which located on chromosome 6q25, was identified to be processed pseudogene. The DNA sequence of *PHBP1* shared the high level of the nucleotide sequence identity (91.3%) with its cognate gene *PHB* [28]. Data from different groups have examined the functional role of *PHB* in human cellular senescence and carcinogenesis [29–32]. Recent findings from Han et al. and Zhong et al. have demonstrated that change in the expression of *PHB*1 was linked to human pancreatic carcinoma and that *PHB* could be used as an early biomarker or a treatment target for pancreatic carcinoma. However, the exact biological functions of *PHBP1* remain unknowns. We investigated the expression level of *PHBP1* in human ESCC tissues and its association with clinicopathological characteristics. Furthermore, we further analyzed its biological functions and precise molecular mechanisms on its cognate gene *PHB* underlying ESCC pathogenesis.

## RESULTS

### Overexpression of *PHBP1* in human ESCC tissues

**Figure 1:**
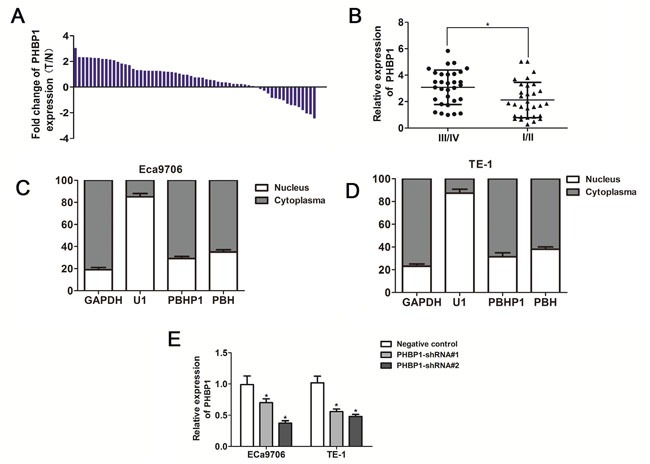
The expression patterns of *PHBP1* in ESCC tissues and cell lines **(A)** *PHBP1* expression in ESCC tissues and adjacent noncancerous tissues. The expression of *PHBP1*was measured by qRT-PCR. GAPDH was used as an internal control. **(B)** Patients with a higher *PHBP1* expression have an advanced stage compared to a lower *PHBP1* expression. Subcellular location of *PHBP1* and *PHB* in Eca9706 **(C)** and TE-1 **(D)** cell lines. GAPDH and RNU1 were used as control of cytoplasm and nucleus, respectively. **(E)** The effectiveness of *PHBP1* knockdown in ECa9706 and TE-1 cells induced by *PHBP1*-shRNAs (shRNA1# and shRNA2#). Relative levels of *PHBP1* RNA expression in ESCC cells were measured by qRT-PCR. Error bars represent the standard deviation (SD) obtained from three independent experiments data; *P<0.05, compared with negative control.

In order to analyze the expression levels of *PHBP1*and *PHB*in ESCC, we performed qRT-PCR on 63 paired ESCC samples and noncancerous samples, and found that levels of *PHBP1*expression was unregulated in 76% (48 of 63), ESCC tissues as compared with that noncancerous samples (*P*<0.01, Figure 1A). Next, we evaluated the correlation between expression levels of *PHBP1*and clinicopathological features of ESCC patients. Remarkably, as showed in Table 1, a significant association between *PHBP1* expression with TNM stage, and patients with high *PHBP1* expression level was significantly correlated with advanced TNM stage in ESCC tissues (Figure 1B).

**Table 1:** Relationship between *PHBP1* and clinicopathological parameters in esophageal squamous cell carcinoma patients

Characteristics	Expression of *PHBP1*		*P*_value_^a^
	Low-*PHBP1* group	High-*PHBP1* group	
**Gender**			
<65	18	22	
≥65	13	9	0.268
**Sex**			
Male	20	16	
Female	11	15	0.305
**Family history of cancer**			
Yes	2	3	
No	29	28	0.640
**TNM stages**			
I+II	17	6	
III+IV	14	25	**0.003**
**Pathological type**			
Highly differentiated	11	13	
Moderately differentiated	13	13	
Low differentiated	7	5	0.493

Furthermore, subcellular location assay revealed that more than 70% *PHBP1* RNA is predominantly located in cytoplasm of Eca9706 and TE-1 cell lines (Figure 1C and 1D; *P*<0.001 for both Eca9706 and TE-1 cells), and small nuclear RNA U6 and GAPDH utilized as control of nucleus and cytoplasm were mostly located in nucleus and cytoplasm, respectively.

### ShRNA-mediated knockdown of *PHBP1* inhibits ESCC cells proliferation and colony formation *in vitro*

**Figure 2:**
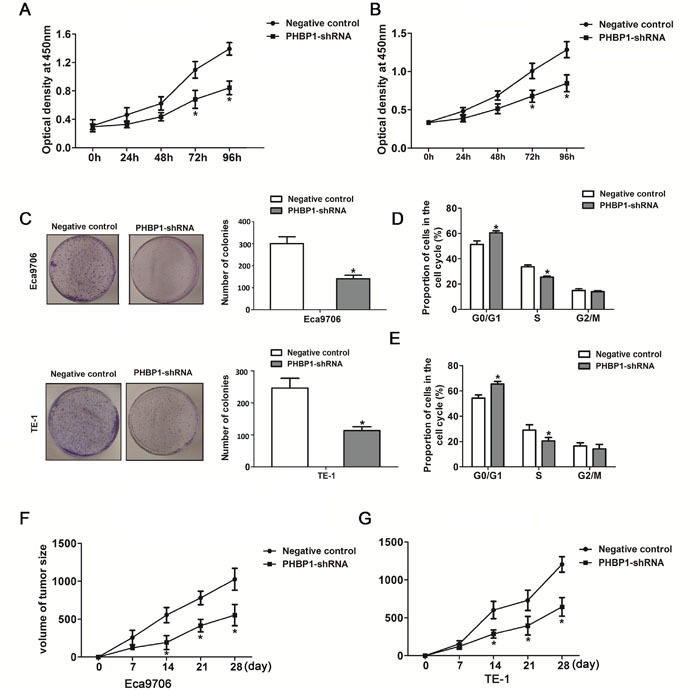
ShRNA-mediated knockdown of *PHBP1* inhibits ESCC cells proliferation and tumor formation of ESCC cells Knockdown of *PHBP1* in Eca9706 cells **(A)** and TE-1 cells **(B)** markedly reduced cell proliferation. Significant proliferation inhibition was observed after 3 days incubation (*P*<0.05). **(C)** Knockdown of *PHBP1* show significant inhibitory effects on the colony formation of ESCC cell lines. The percentage of Eca9706 cells **(D)** and TE-1 cells **(E)** with stable knockdown of *PHBP1* in S phase was significantly decreased, compared to negative control. **(F** and **G)** Tumor growth in nude mice subcutaneously injected into flanks with *PHBP1*-shRNA or negative control. Data are presented as means± SDs (n=8/group). The values was present as means ± SD. Statistical analyses were performed using One-way ANOVA or two-tailed Student's t-test, *P<0.05, compared with negative control.

We developed a downexpression of *PHBP1* model in ESCC cells using lentiviral transduction to test whether *PHBP1* was functionally involved in ESCC tumorigenesis. The inhibition of *PHBP1* in ECa9706 and TE-1 cells induced by *PHBP1*-shRNAs (shRNA1# and shRNA2#) was confirmed by qRT-PCR with the scramble shRNA served as the negative control. Because of their effectiveness, we utilized *PHBP1*-shRNA2# transfected cells as the stable cells with knockdown of *PHBP1* (Figure 1E). CCK-8 assays and colony formation assays were used to detect the impact of *PHBP1* knockdown on proliferation of the ESCC cell lines. ECa9706 and TE-1 cells with the stable knockdown of *PHBP1* lead to a significantly decreased cell growth by more than 42% and 47% relative to negative control at day 4 in both cell lines, respectively (*P*<0.05 in ECa9706 and *P*<0.05 in TE-1; Figure 2A and 2B). Similarly, the capacity of stable knockdown of *PHBP1* to form colonies was reduced by 52% in ECa9706 cell and 61% in TE-1 cells (*P*=0.002 in ECa9706 and *P*=0.001 in TE-1; Figure 2C).

### Knockdown of *PHBP1* significantly induced cell-cycle arrest at the G1-G0 phase in ESCC cells

To further evaluate whether the functional consequences of downregulation of *PHBP1* was induced by cell cycle, flow cytometry assay was performed. Compared to the negative controls, inhibition of *PHBP1* led to a significant accumulation of cells at G0/G1-phase (60.49%± 1.62% vs 51.36% ± 2.84% in ECa9706 and 65.47%± 2.00% vs 54.35% ± 2.60% in TE-1; Figure 2D) and a markedly decrease of cells in S-phase (25.56%± 0.84% vs 33.65% ± 1.52% in ECa9706 and 20.41% ± 2.78% vs 29.12%± 2.14% in TE-1; Figure 2E). Taken together, the results imply that *PHBP1* may inhibited ESCC cell proliferation by preventing cell-cycle progression through S-phase.

### Inhibition of *PHBP1* leads to reduced tumor growth in nude mice

An animal experiment was further used to confirm the effect of *PHBP1* on tumorigenesis *in vivo*. ESCC cells with stable expression of *PHBP1*-shRNA or negative control was subcutaneously injected into the nude mices, respectively. The tumor was measured every 3 days. As showed in Figure 2F and 2G, during 4 weeks follow up measurement, the initiation and growth of tumor formed in mices with the inhibition of *PHBP1* cells were significantly slower than that tumor formed in mices with negative control cells (*P*<0.05 for both Eca9706 and TE-1 cells).

### Coordinated expression of *PHBP1* and *PHB* in ESCC cell lines

**Figure 3:**
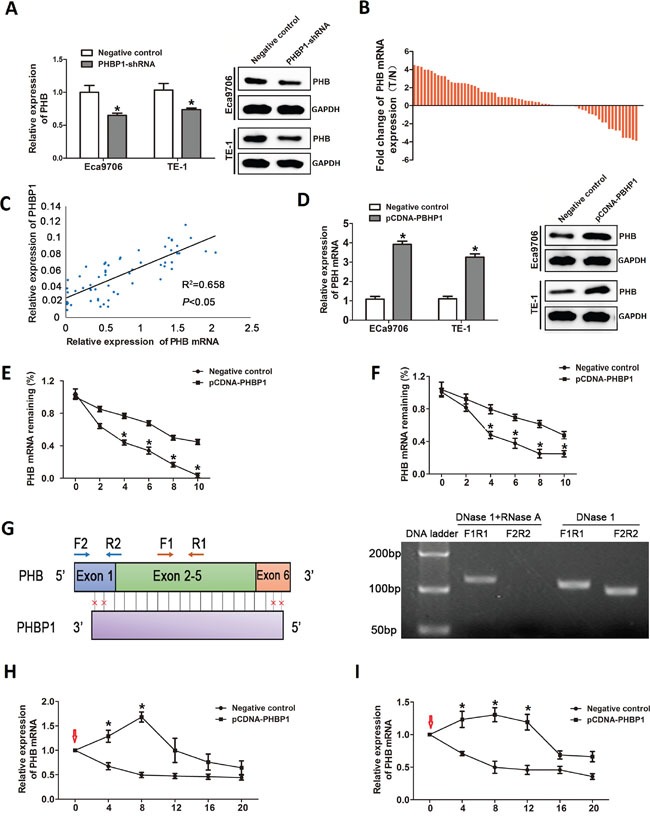
The regulatory effect of *PHBP1* on *PHB* expression **(A)** The *PHB* mRNA levels (left) and protein levels (right) in both Eca9706 and TE-1 cells after *PHBP1* knockdown by qRT-PCR analysis and western blot analysis. **(B)** The expression of *PHB* was measured by qRT-PCR in ESCC tissues and adjacent noncancerous tissues and normalized to GAPDH. Statistical differences between groups were performed using the two-tailed Student's t-test (*P*<0.05). **(C)** *PHBP1* and *PHB* expression levels were positively correlated in ESCC tissues (R^2^= 0.658, *P*<0.05 by Pearson correlation test). **(D)** The *PHB* mRNA levels (left) and protein levels (right) in both Eca9706 and TE-1 cells transfected with pCDNA-*PHBP1* or negative control. The *PHB* mRNA half-life by incubating Eca9706 cells **(E)** and TE-1 **(F)** transfected with pCDNA-*PHBP1* or negative control with actinomycin D using qRT-PCR. **(G)** *PHBP1*transcript overlaps with 90% nucleotides of *PHB* mRNA indicated by vertical black lines (left). The blocks with colors indicate exons; Primer sites (F1: forward primer 1 and R1: reverse primer 1) in *PHB* mRNA represents the non-overlapping region between *PHBP1* and *PHB* transcripts, and primer sites (F2: forward primer 2 and R2: reverse primer 2) indicates the overlapping region between *PHBP1* and *PHB* transcripts. RT-PCR data depicted the detection of *PHBP1* formed RNA duplex with *PHB* mRNA that protected *PHB* mRNA from RNase degradation (right) which digested the single-stranded RNA. PCR products were amplified using F1R1 and F2R2. The stability of *PHB* mRNA was examined by incubating Eca9706 cells **(H)** and TE-1 **(I)** transfected *PHBP1* after 24h with α-amanitin (30mM) for 4, 8,12,16 and 24h compared with cells transfected with an empty vector; the red arrows indicated the time of α-amanitin added into the medium. Data are presented as mean±SD based on at least three independent experiments. *P<0.05, compared with negative control. Statistical analyses were performed using One-way ANOVA or two-tailed Student's t-test.

Considering the special complementarily sequence of the *PHBP1* gene and its cognate gene *PHB* at the nucleotide level, it attracted our attention to sought to elucidate the effect of *PHBP1* on its cognate coding gene RNA. We first delineated *PHBP1* and *PHB* expression patterns in ESCC cells with stable expression of *PHBP1*-shRNA. The result showed that *PHB* mRNA and protein levels were reduced in ECa9706 cells, after knockdown *PHBP1* expression by *PHBP1*-shRNA. The expression patterns of *PHBP1* and *PHB* was confirmed in TE-1 cells with stable expression of *PHBP1*-shRNA (Figure 3A). Based above data, we further examined the location of *PHB* following cell fractionation, and the results showed that *PHB* is mostly localized in cytoplasma (>65 %), similar to the subcellular location of *PHBP1* (Figure 1C and 1D).

### Expression patterns of *PHB* mRNA in ESCC tissue samples

Moreover, we also detected the expression levels of *PHB* in the same cohort of 63 ESCC tissue sample as described above. Similarly, *PHB* mRNA expression levels was significantly higher in 80% (36 of 41) tumor tissues than in noncancerous samples (*P*<0.05, Figure 3B). Interestingly, we found a similar concordant expression pattern of *PHBP1* and *PHB* mRNA levels in the same samples, and *PHBP1* levels and *PHB* mRNA levels were positively correlated in ESCC tissues (R^2^= 0.658, *P*<0.05; Figure 3C). These results implied that *PHBP1* may activate *PHB* expression at both the RNA and protein levels.

### The RNA stability of *PHB* was increased by *PHBP1*

We measured the *PHB* mRNA half-life by incubating cells with actinomycin D using qRT-PCR. The results showed that the transcript level and protein level of *PHB* was significantly increased in ESCC cells with *PHBP1* overexpressed, compared with the controls (Figure 3D). Furthermore, the half-life of *PHB* mRNA was prolonged from 2.5 to 8h in ECa9706 cells and from 4 to 9h in TE-1 cells after actinomycin D treatment than in control cells (Figure 3E and 3F). These results imply that *PHBP1* could increase *PHB* mRNA stability.

### *PHBP1* controls *PHB* mRNA stability by *PHBP1*/*PHB* duplex formation

Currently, as reported in several studies, numerous AS lncRNAs strongly co-expressed with their cognate mRNAs through forming duplex complexes, which is protected from ribonuclease resistant [33]. In addition, because of the perfectly complementary regions between the 90% nucleotides for *PHBP1* and *PHB* mRNA (Figure 3G), we determined RNase protection assay (RPA) to explore whether *PHBP1* could upregulates *PHB* transcription through forming a ribonuclease-resistant *PHBP1*/*PHB* protective duplex. As shown in Figure 3G, RT-PCR data using primers F1R1 (F1: forward primer 2 and R1: reverse primer 1) located the overlapping region between *PHBP1* and *PHB* transcripts depicted the detection of *PHBP1* formed RNA duplex with *PHB* mRNA that protected *PHB* mRNA from RNase A degradation. We further used the transcriptional inhibitor, α-amanitin to investigate the effect of *PHBP1* on the stabilization or augmentation of *PHB* expression. Subsequent RT-PCR data revealed that *PHB* stability in ECa9706 and TE-1 cells overexpressing *PHBP1* was increased after treated with α-amanitin (30mM) at 0h compared with cells transfected with an empty vector (Figure 3H and 3I), while RNA polymerase II expression was downregulated. Taken together, these data infer a role for *PHBP1* in the stabilization of *PHB* expression by *PHBP1* RNA/*PHB* mRNA heteroduplex formation of their perfectly complementary regions.

## DISCUSSION

*PHB* gene, is a member of evolutionarily conserved family of membrane proteins, which plays essential roles in the regulation of human various pathophysiological processes and various cancers [34–36]. The role and the prognostic significance of *PHB* expression patterns suggest that it could be as a potential biomarker in human diseases, including particularly human cancers. *PHBP1*, which is transcribed in antisense orientation with respect to *PHB* and shared the high level of the nucleotide sequence identity with its cognate gene *PHB*. However, to date, the expressions and functions of *PHBP1* in ESCC physiological functions remain obscure.

In this study, we investigated *PHBP1* and *PHB* expression in an independent cohort of ESCC tissues and normal tissues. We found that the expression of *PHBP1* and *PHB* were both higher in ESCC tissues, and *PHBP1* expression was positively correlated with *PHB* in ESCC tissues. Clinical analysis showed that ESCC patients with higher *PHBP1* expression tend to have advanced TNM stage. These findings indicate the abnormal expression of *PHBP1* is linked to ESCC carcinogenesis.

aslncRNAs as *one* class *of* important heterogeneous lncRNAs transcribed in the opposite indirection with respect to one protein coding gene [37, 38]. They are defined as being *complementary to one* or *more* messenger RNA molecules [39]. Mounting lines of evidence have demonstrated that aslncRNAs have played essential regulatory roles in various biological processes [40, 41]. A growing studies also supports their importance in carcinogenesis and cancer development. So far, it is widely studied that aslncRNAs can activate or inhibit the expression of complementary coding genes at chromatin, *transcriptional and post- transcriptional* levels [42–45]. One example including promotion of the aggressive behaviors of colorectal carcinoma cells by FEZF1 antisense RNA1 (FEZF1-AS1), showed that FEZF1-AS1 could increase its corresponding cognate gene mRNA FEZF1 through regulating the transcription or mRNA stability of FEZF1 [26]. In this study, we investigated the biological functions of *PHBP1* on ESCC tumorigenesis and the regulation mechanisms on its cognate gene *PHB*. Based on our results, we found that *PHBP1* could activate its corresponding sense gene *PHB*. Mechanistically, actinomycin D assay provided evidences that *PHBP1* prolonged *PHB* mRNA half-life. In addition, RPA further confirmed that *PHBP1* could interact with *PHB* mRNA forming RNA duplex compound to induce *PHB* transcription and translation. Furthermore, subcellular location assay may provide clues regarding the possible molecular mechanism of *PHBP1* exerting its biological function in cytoplasm as *PHBP1* was predominantly located in cytoplasm of ESCC cell lines, similar to *PHB*. Both *in vitro* and *in vivo* data showed that the silence of *PHBP1* induced cell cycle arrest at the G0–G1 phase and significantly inhibited proliferation and tumor growth of ESCC cells. Taken together, the effects of *PHBP1* in aggressive phenotypes of ESCC, at least in part, dependent on the induction of *PHB*.

In summary, we provided a better understanding of the biology of ESCC carcinogenesis by *PHBP1*. We investigate that the *PHBP1* was significantly upregulated in ESCC tissues and increased expression of *PHBP1* might play a promotion role in ESCC carcinogenesis by binding to *PHB* mRNA forming RNA duplex, consequently inducing *PHB* mRNA stability and transcription. All of these findings illustrate the important roles of *PHBP1* in ESCC carcinogenesis and the potential role of *PHBP1* as a novel biomarker for ESCC.

## MATERIALS AND METHODS

### Cell culture

The human embryonic kidney cells 293T and the human ESCC cell lines (ECa9706 and TE-1) were purchased from the Chinese Academy of Sciences (Shanghai, China), cultured in DMEM or RPMI 1640 medium with 10% fetal bovine serum (HyClone, Logan, USA), respectively. These cells were maintained at 37 °C in a humidified chamber with 5% CO_2_.

### Animals’ model

4–6 weeks old female nude mices (20-25g/each) were obtained from Chinese Academy of Science Shanghai Experimental Animal Center and were maintained with food and water ad libitum. Protocol for animal experimentation was carried out in strict accordance with the laboratory animal care guidelines of Laboratory Animals of the National Institutes of Health.

### Tissue preparation

63 paired ESCC tissues and adjacent normal tissues were collected from patients who were diagnosed with ESCC at Cancer Hospital, Peking Union Medical College and used in the investigation of clinicopathological and functional role of *PHBP1*. None of these subjects have received any treatment of ESCC, such as chemotherapy or radiotherapy before surgical resection preoperative. A comprehensive set of clinicopathological data were recorded, including age, gender, size of primary tumor, tumor differentiation and TNM stage. The clinical characteristics are summarized in Table 1. The stage of disease was determined according to the TNM classification system. The ages of all the patients ranged from 29 to 83 years, with a median age of 65 years and each patient signed the informed consent before donating the tissue specimens. And the study was also approved by the ethics committee of Cancer Hospital, Peking Union Medical College.

### RNA isolation and quantitative real-time PCR (qRT-PCR)

Total RNA was isolated using TRIzol reagent (Invitrogen). For qRT-PCR, RNA (1μg) was used for cDNA synthesis by using a Reverse transcription kit (Takara, Dalian, China) according to the manufacturer's instructions. qRT-PCR analyses were carried out with SYBR Premix Ex Taq (Takara, Dalian China) in the ABI 7500 RT-PCR system (Applied Biosystems, Foster City, USA). Thermocycling parameters: 95°C for 20s followed by 45 cycles of 95°C for 10s and 60°C for 30s. The data was converted to fold change normalized to the expression level of *GAPDH*, which served as an endogenous control. For *PHB* mRNA stability analysis by qRT-PCR, 18s ribosomal RNA, a product of RNA polymerase I, was used as an internal control.

### Subcellular fractionation

The procedure of nuclear and cytosolic fractions from ESCC cells were performed based on the manufacturer's instructions using the PARIS Kit (Life Technologies, Carlsbad, CA, USA). The resulting supernatants and pellets were collected for the cytosolic fraction and nuclear fraction, respectively. Subsequently, the RNAs of cellular compartments were sequentially extracted and expression of *PHBP1* or *PHB* was quantified by qRT-PCR.

### Vector preparation and transfection of ESCC cells

The lentiviral vector PLVX containing *PHBP1*-shRNA (1# and 2#) or scrambled control sequence were synthesized by GenPharma (Shanghai, China) and were then packaged with pPACKH1 Lentivector Packaging Plasmid mix (System Biosciences) into 293T cells. Typically, ECa9706 cells and TE-1 cells were seeded at six-well plates for lentiviral transduction. Infected ESCC cells were selected by G418 to gain ESCC cells with stable knockdown of *PHBP1*. QRT-PCR was used to quantify the knockdown efficiency of *PHBP1*.

### Western blotting analysis

The western blotting analysis procedures were performed as described as follows. Briefly, Total proteins extracted from transfected cells were subjected to the 10% SDS-polyacrylamide gel electrophoresis (SDS-PAGE) and then transfer to the nitrate cellulose (NC) membranes. The membrane was probed with specific antibody PHB overnight at 4°C, which were purchased from Cell Signaling Technology, Inc (CST). GAPDH antibody was used as control.

### Actinomycin D assay

To detect the effect of *PHBP1* on the stability of *PHB* mRNA, the *PHBP1* transcript was synthesized and then subcloned into a pCNDA-3.1 vector (Invitrogen, Shanghai, China). For the Actinomycin D assay, the ESCC cells were planted in six-well plate before transfection. When ESCC cells were about 70% confluent, the pCDNA-*PHBP1* or empty vector was transfected into ESCC cells using Lipofectamine 2000 reagent. After 24h, the transfected ESCC cells were cultured with Actinomycin D (Sigma) which was used to suppress transcription. After 2, 4, 6, 8 and 10h treatment, the cells were harvested and extracted total RNA to detect the half-life of *PHB* induced by *PHBP1*. The final concentration of actinomycin D was used at 5μg/ml.

### RNase protection assay

Considering the overlapping region of the *PHBP1* and its cognate transcript *PHB*, we performed RNase protection assay to test whether *PHBP1* can form the RNA duplex with its cognate sense RNA, Briefly, total RNAs from ESCC cells transfected pCDNA-*PHBP1* or empty vector were extracted and treated with the RNase A+T cocktail (Ambion) which digested the single-stranded RNA with increasing amounts. The remaining RNA duplexes were subjected to RT-PCR to detect *PHBP1* and *PHB* employing two sets of primers to target the overlapping and non-overlapping part of *PHBP1* and *PHB* transcripts.

### Cell proliferation assays

For cell viability analyses, ECa9706 and TE-1 cells with stable knockdown of *PHBP1* were plated in a 96-well plate at 2000 cells per well, maintained in RPMI1640 containing 10% FBS for 1, 2, 3 and 4 days. CCK-8 kit (Dojindo Laboratories, Kumamoto, Japan) were conducted to measure the absorbance of the cells at OD 450nm every 24h, according to the manufacturer's instruction. The relative Cell viability rate was normalized with the value at day 0. All experiments were performed in triplicate. For colony formation assay, the indicated stable cells are seeded out in appropriate numbers in 60mm plates to form colonies. After two weeks, cell colonies are fixed with methanol, stained with 0.5% crystal violet and were counted normalized with the controls.

### Cell cycle analysis

For cell cycle analysis, the indicated ESCC cells with stable overexpressed of *PHBP1* were harvested and fixed with 70% ethanol. After the cells were stained with propidium iodide (Life Tecnologies) following the protocol, DNA content of the cells in G0/G1, S, and G2/M phase were qualified on FACS Calibur Flow Cytometer (BD Biosciences).

### Xenograft experiment

For the *in vivo* tumor growth assay, a total of 100μL with 6×10^6^ suspended ECa9706-shRNA-*PHBP1*, TE-1-shRNA-*PHBP1* or shRNA-NC cells were injected into either side of the posterior flank of 5 weeks old BALB/c nude mices. Seven days after injection, the volumes of the subcutaneous tumor were measured every 3 days using the equation Volume=length×width^2^×0.5.

### Statistical analyses

All statistical analyses were performed using SPSS software (SPSS, Chicago, Illinois, USA). All cell biology assays performed in at least three independent experiments. Two-tailed Student's t-test or One-way ANOVA was used to assess the differences between variables among the groups. Pearson correlation test was performed to evaluate the association between the level of *PHBP1* expression and *PHB* expression level. The data of experiments were presented as mean ± standard deviation (SD). *P*<0.05 was noted as statistical significance.
